# Is Blackwater Fever a Form of Malaria?

**Published:** 1908-11-14

**Authors:** 


### TROPICAL DISEASES.

### IS BLACKWATER FEVER A FORM OF MALARIA?

THE relations of blackwater fever to malaria upon the one hand and to quinine upon the other are points upon which there has been the most heated discussion from time to time, and it cannot be said that either question has yet been entirely settled. It is of interest, however, to consider the opinions and arguments of those who have had opportunities of studying blackwater fever over a period of several recent years, aided by the microscope and by all the modern improvements in the technique of blood examinations and in the knowledge of the special points upon which a watch must be kept.

We have recently (Oct. 17, 1908, page 62) given an account of the Hearsey treatment of blackwater fever, and of the immense reduction in the mortality from the disease when this treatment is employed. Not only, however, are modern methods successful in curing the illness in a way that was impossible a score of years ago, but also it is probable that the disease can be prevented by the adoption of suitable precautions *ab initio*. Captain Hardy is strongly of the opinion that blackwater is a complication of malignant tertian malaria—a complication comparable to hyperpyrexia, coma, or any of the other well-known symptoms of danger that malignant malaria may give rise to.

### CAPTAIN HARDY'S ARGUMENTS.

The argument which Captain Hardy adduces in favour of blackwater fever being a complication of malignant tertian fever may be briefly summarised as follows:—

1. Blackwater fever is invariably preceded by malignant tertian fever; usually a sequence of relapses, occasionally a low continuous fever.

2. Parasites can be found in the peripheral blood in every case, not at the height of the black-urine stage, but in the pre-hæmoglobinuric phase, and to a lesser extent during the first few hours of the illness. Stephens states that of ninety-five cases examined by competent observers 95.6 per cent. had parasites on the day before the attack. Captain Hardy found malignant tertian parasites to be present in numbers in the pre-hæmoglobinuric stage, and he was able to observe these parasites, abundant a short while before, disappear within an hour or so after the appearance of the hæmoglobinuria. He suggests that failure to find them during the illness may possibly be due to the hæmolysis, which occurs so rapidly, affecting the weakest—that is to say, the infected—corpuscles first.

3. Blackwater fever may occur in a malarial patient who has left an endemic district some time since, and even after his return to England. This is precisely analogous to the relapses of malaria itself.

4. In cases of blackwater fever the differential leucocyte count in blood films shows a high percentage of large mononuclear cells, averaging 20 per cent. or more. This high percentage of large lymphocytes is only known to occur in malaria.

5. Indian natives—Sikhs and traders—do not suffer from the disease as frequently as Europeans, the relative incidence liability being, roughly, as 1 to 4. This appears to point to a partial immunity to the disease in the case of Indian natives. Amongst the millions of African natives extremely few cases have been reported. As these natives all suffer from a chronic malignant tertian infection in childhood, it would appear that their freedom from blackwater fever may be connected with that fact. Captain Hardy has collected statistics which show that healthy native children on Lake Nyassa, under seven years of age, showed malignant tertian parasites in their blood to the extent of 75 per cent. Moreover, natives from America and the West Indies suffer almost as badly as Europeans. Amongst the latter the liability to attack by blackwater fever appears to diminish after five years' residence.

6. Blackwater fever, as regards its geographical distribution, follows that of malignant tertian fever very closely.

7. Systematic quinine takers enjoy a very high immunity from attack. Plehn's figures in Clifford Allbutt's "System of Medicine" show this very plainly.

Captain Hardy further expresses the opinion that the "irregular" taking of quinine is to be highly condemned, being responsible for most, if not all, of the attacks of quinine hæmoglobinuria. The method of taking quinine varies: many prefer to take 5 grains daily; another plan is to take 15 grains every sixth or seventh day. The latter method is becoming widely adopted, and with great success.

It is not every form of "malaria" that is liable to be complicated by blackwater. At least three kinds of malaria have to be distinguished, namely, benign tertian, quartian, and malignant tertian. It is the latter which is followed by the hæmoglobinuria (blackwater) as a complication, and the prophylaxis of the complication is therefore simply that of the antecedent disease.

### THE SALARIES OF MEDICAL OFFICERS.

THE Elementary Education Sub-Committee of the Derby Education Committee recently resolved to advertise in the medical papers for an assistant medical officer at a salary of £200 per annum. The *British Medical Journal*, however, declined to insert the advertisement on the ground that in the opinion of the British Medical Association these whole-time appointments should receive a minimum salary of £250 per annum. The manager of the *Lancet* also declined to publish the advertisement, in consequence of representations from local medical men. At a subsequent meeting of the Sub-Committee, it was proposed that the advertisement should be inserted in the lay Press, whilst another proposal was that the town should be divided into sections and the work offered to local medical men. These suggestions were, however, rejected in favour of a resolution to conform to the opinion of the British Medical Association, and to make the salary £250 per annum.

